# A Spontaneous Model of Experimental Autoimmune Encephalomyelitis Provides Evidence of MOG-Specific B Cell Recruitment and Clonal Expansion

**DOI:** 10.3389/fimmu.2022.755900

**Published:** 2022-02-03

**Authors:** Florent Salvador, Laure Deramoudt, Frédéric Leprêtre, Martin Figeac, Thomas Guerrier, Julie Boucher, Mathilde Bas, Nathalie Journiac, Anneli Peters, Lennart T. Mars, Hélène Zéphir

**Affiliations:** ^1^ Univ. Lille, Inserm, CHU Lille, Laboratory of Neuroinflammation and Multiple Sclerosis (NEMESIS), UMR-S1172, Lille Neuroscience & Cognition, LICEND, FHU Imminent, Lille, France; ^2^ UMS2014-US51, Genomics and Structural Platform, Lille University, Lille, France; ^3^ Univ. Lille, Inserm, CHU Lille, U1286, INFINITE-Institute for Translational Research in Inflammation, Lille, France; ^4^ Institute of Clinical Neuroimmunology, Hospital and Biomedical Center of the Ludwig-Maximilian University (LMU), Martinsried, Germany; ^5^ CRC-SEP of Lille, CHU of Lille, Lille, France

**Keywords:** B cell, repertoire, autoimmunity, experimental autoimmune encephalomyelitis (EAE), multiple sclerosis, myelin oligodendrocyte glycoprotein (MOG), TCR^1640^ transgene mouse model, demyelinating antibodies

## Abstract

The key role of B cells in the pathophysiology of multiple sclerosis (MS) is supported by the presence of oligoclonal bands in the cerebrospinal fluid, by the association of meningeal ectopic B cell follicles with demyelination, axonal loss and reduction of astrocytes, as well as by the high efficacy of B lymphocyte depletion in controlling inflammatory parameters of MS. Here, we use a spontaneous model of experimental autoimmune encephalomyelitis (EAE) to study the clonality of the B cell response targeting myelin oligodendrocyte glycoprotein (MOG). In particular, 94% of SJL/j mice expressing an I-A^s^: MOG_92-106_ specific transgenic T cell receptor (TCR^1640^) spontaneously develop a chronic paralytic EAE between the age of 60-500 days. The immune response is triggered by the microbiota in the gut-associated lymphoid tissue, while there is evidence that the maturation of the autoimmune demyelinating response might occur in the cervical lymph nodes owing to local brain drainage. Using MOG-protein-tetramers we tracked the autoantigen-specific B cells and localized their enrichment to the cervical lymph nodes and among the brain immune infiltrate. MOG-specific IgG1 antibodies were detected in the serum of diseased TCR^1640^ mice and proved pathogenic upon adoptive transfer into disease-prone recipients. The ontogeny of the MOG-specific humoral response preceded disease onset coherent with their contribution to EAE initiation. This humoral response was, however, not sufficient for disease induction as MOG-antibodies could be detected at the age of 69 days in a model with an average age of onset of 197 days. To assess the MOG-specific B cell repertoire we FACS-sorted MOG-tetramer binding cells and clonally expand them *in vitro* to sequence the paratopes of the IgG heavy chain and kappa light chains. Despite the fragility of clonally expanding MOG-tetramer binding effector B cells, our results indicate the selection of a common CDR-3 clonotype among the Igk light chains derived from both disease-free and diseased TCR^1640^ mice. Our study demonstrates the pre-clinical mobilization of the MOG-specific B cell response within the brain-draining cervical lymph nodes, and reiterates that MOG antibodies are a poor biomarker of disease onset and progression.

## Introduction

B cells are essential components of the immune infiltrate that characterizes active demyelinating lesions in multiple sclerosis (MS). The histopathology of patients with relapsing-remitting MS, commonly points to the implication of the humoral response. The localization of plasma blasts and B cells in the perivascular cuffs are associated with the presence of antibodies, activated complement and Fc-receptor expressing myeloid cells indicative of antibody mediated tissue damage (1). The clinical relevance of antibodies is further underlined by the diagnostic value of oligoclonal bands (OCBs) that are present in the cerebrospinal fluid (CSF) of 90% of MS patients. Comparison of transcriptomes of CSF B cells and CSF Ig proteomes revealed that clonally expanded B cells in the CSF produce OCBs (2). Evidence of somatic hypermutation indicates that this is an antigen-driven process (3, 4), which occurs in the brain-draining cervical lymph nodes and possibly in the meningeal ectopic lymphoid structures (5, 6). The specificity of this adaptive immune response is composite and evolves over time (7–9).

Disease progression and worsening are similarly thought to be influenced by the humoral response. The transition to secondary progressive MS (SPMS) is associated with the accumulation of B cells in the leptomeningeal space covering the cortical surface. These ectopic B cell follicles share immunological traits with germinal centers and are thought to contribute to subpial demyelination (10–13). The detrimental nature of the B cell response was formally demonstrated by the therapeutic efficiency of B-cell depletion for both relapsing-remitting (RR) and progressive MS (14–16). This pivotal demonstration is of particular interest as this strategy targets CD20^+^ B cells thus sparing CD20 negative plasmocytes. In MS this results in clinical improvement without reducing IgG titers, nor by impacting plasmocyte frequency implicating a cellular role for B lymphocytes (17).

During the germinal center reaction B cells present antigen to follicular helper T cells in order to control the process of somatic hypermutation (18, 19). Antigen uptake is achieved *via* their B cell receptor (BCR) allowing the internalization, processing and presentation of the captured protein to T cells (20). This route of specific antigen uptake might have larger implications in the context of chronic inflammatory diseases such as MS. Indeed, a selective deficiency of MHC-II in B cells was demonstrated to cause resistance to experimental autoimmune encephalomyelitis (21). Mice expressing the heavy chain of a MOG-specific BCR (IgH^MOG^) were compared to a novel IgH^MOG^ variant in which the secretion of transgenic MOG-antibodies is invalidated. Both genotypes remained fully susceptible to recombinant human MOG (rhMOG)-induced EAE, formally demonstrating that among transgenic MOG-specific B cells the ability to secrete antibodies did not modify their pathogenicity (21). In MS patients, circulating B cells strongly express MHC II accompanied by the induction of costimulatory molecules (22, 23). These memory B cells can present myelin-derived peptides to T cells, leading to pathogenic T cell activation and CNS homing (24, 25). Their impact on the T cell response is variable. The prominent production of IL-6 by B cells and the reduced magnitude of Th1 and Th17 responses in their absence point to an inflammatory role in MS (21, 26–28). This impact is extended to myeloid cells as the production of GM-CSF by memory B cells favors their differentiation (29). Of similar importance is the discovery that inflammatory B cells are balanced by the induction of regulatory B cells able to reduce the magnitude of inflammation through production of IL-10 and IL-35 (30–32). Plasma cells are similarly endowed with this regulatory capacity (33). Single cell RNA sequencing of CSF B cells in MS revealed clonally related B cells with a pro-inflammatory profile and downregulation of the SMAD/TGF-β1 pathway (34). This B cell cytokine profile might be predictive as in patients with clinically isolated syndrome (CIS) the evolution to MS was associated with a profile favoring IL-6 concomitantly with a loss of IL-10 production (35).

Spontaneous models of EAE provide a complementary paradigm to study myelin-specific B cells over the course of CNS autoimmune disease. Spontaneous EAE develops in the TCR^1640^ transgenic SJL/j mice owing to the expression of an I-A^s^ restricted TCR specific for the MOG peptide 92-106 (MOG_92-106_) on 70% of CD4 T cells (36). Over time, nearly all TCR^1640^ develop inflammatory demyelinating lesions in the brain, optic nerves and spinal cord, reminiscent of the active lesions characteristic of MS. Priming of the adaptive immune response implicates the microbiota given that axenic TCR^1640^ mice refrain from developing clinical or histopathological EAE. In gnotobiotic TCR^1640^ mice, naïve CD4 T cells are likely to be activated within the gut-associated lymphoid tissue (GALT) and re-stimulated within the brain draining cervical lymph nodes by their cognate myelin antigen (37). The microbiota can be instructive to the encephalitogenic immune response as transplanting axenic TCR^1640^ mice with the microbiota of homozygous twins discordant for MS allowed for autoimmune demyelination only in mice receiving the microbiota of the diseased sibling (38).

The TCR^1640^ mice provide an interesting opportunity to study the mobilization of the humoral response. Importantly, in these mice effector B cells are recruited from a B cell repertoire that is non transgenic and unmanipulated. Germinal centers are detected in the cervical lymph nodes of young RR mice prior to disease onset, implying that TCR^1640^ follicular helper T cells drive the isotype switching and affinity maturation of the MOG-specific B cell response (37). This B cell response is pathogenic as B cell depletion using anti-CD20 monoclonal antibodies prevents the development of spontaneous EAE in the TCR^1640^ mice (36). In this study we used recombinant MOG tetramers to trace the autoantigen-specific B cell response in the TCR^1640^ mice, which developed an acute EAE with late onset in our animal facility. Single B cell expansion provided evidence of a B cell clonotype that emerges over the course of spontaneous autoimmunity in the TCR^1640^ mice.

## Methods

### Mice

Heterozygous TCR^1640^ SJL/j (36), homozygous IgH^MOG^ C57BL/6 mice (39), and heterozygous C57BL/6 2D2 mice (40) were housed under specific pathogen-free (SPF) conditions at the animal facility of the University of Lille which is accredited by the French Ministry of Agriculture to perform experiments on live mice in appliance to the French and European regulations on care and protection of the Laboratory Animals (EC Directive 2010/63). Water and food was provided *ad libitum*, hydrogels (Bioservice, Uden, The Netherlands) were added twice weekly. All experimental protocols were approved by the local ethics committee and and the Ministère de l’Enseignement Supérieur de la Recherche et de l’Environnement (5157-2016111011562655) in compliance with European Union guidelines.

### EAE Scoring and Induction by Serum Transfer

EAE severity was scored as follows: 0, healthy; 1 for tail atony; 2 for hind limb weakness; 3 for hind limb paralysis; 4 for quadriplegia; 5 for moribund after at least 2 consecutive days of clinical disease. For serum transfer EAE, sera were transferred intravenously, *via* retro-orbital injection in disease-prone 2D2 TCR transgenic recipients. Each recipient received the serum of a single, distinct, donor. On day 0, 2D2 mice received 200 ng of pertussis toxin (List Biological Laboratories, Campbell, CA) intravenously by retro-orbital injection. Two days later a second i.v. injection of pertussis toxin was accompanied by either 50 µL of serum from disease-free TCR1640, diseased TCR1640 or NTL mice or either 50 µL of PBS.

### Cell Isolation

Single-cell suspensions were prepared from spleen and cervical lymph nodes by mechanical disruption *via* forcing through 40-µm cell strainers. B-cells were purified using a mouse B-cell isolation kit (EasySTepTM Mouse B cell Isaolation Kit, Stemcell, Köln, Germany). For the isolation of brain infiltrating cells mice were anesthetized with ketamine and transcardially perfused with cold PBS. Brain and spinal cord were collected separately, homogenized and digested with 2 mg/ml collagenase D, 20 μg/ml DNase I, 1 μg/ml TLCK (Roche, Basel, Switzerland) for 30-45 min at 37°C. Adding 60 mM of EDTA stopped the reaction. Cells were then washed, resuspended in 37% of Percoll, layered on 70% Percoll and overlaid with 30% Percoll. After a 20-minute centrifugation at 2000 rpm the mononuclear cells were collected from the interface.

### rMOGm Monomer and Tetramer Production

Polyethylenimine linear (PEI, CliniSciences, Nanterres, France) treated HEK cells were transfected with a pTT5 plasmid encoding for a histidine and Avi tagged MOG_1-125,_ according to the protocol described in (41). Transfection efficiency of 21% was determined using a GFP plasmid. After 7 days of culture, supernatants were collected and rhMOG was purified by affinity chromatography using His TrapTM HP columns (Cytivia, Marlborough, MA). rMOG was eluted using an imidazole gradient (Sigma-Aldrich), followed by dialysis against PBS. A single biotin molecule was attached to the AviTag using the biotinylating kit (BirA500 kit, Avidity, Aurora, CO). The single-biotin rMOGm monomer was tetramerized at a ratio of 4mol of biotin rMOGm monomer to 1 mol of FITC or PE-conjugated streptavidin (Biolegend, San Diego, CA). The rMOGm tetramer that we refer to as MOG_tet_ was validated by flow cytometry using splenocytes from IgH^MOG^ mice. Validated MOG_tet_ labelled at least 70% of MOG-specific B cells present among IgH^MOG^ splenocytes.

### Flow Cytometry and FACS Sorting

For detection of cell surface markers, cells were stained in FACS buffer (PBS containing 1% BSA and 0.1% NaN_3_) with fluorochrome-labelled monoclonal antibodies: APC-Cy7-conjugated anti-CD4 (RM4-5) or BV786-conjugated anti-CD4 (RMA-5], PE-Cy5-conjugated anti-CD19 (6D5) or Pacific-Blue-conjugated anti-CD19 (6D5), Pacific-Blue-conjugated anti-CD38 (6D5), Alexa Fluor 647-conjugated anti-GL-7 (GL7), PE-conjugated anti-CD138 (281-2), FITC-conjugated anti-IgM^b^ (AF6-78), APC-conjugated anti-CD45.1 (A20), PE-conjugated anti-CD45.2 (104); PerCP-Cy5-conjugated anti-B220 (RA3-6B2), BV-605-conjugated anti-CD19 (1D3), PE-Cy7-conjugated anti-FAS (Jo2), or Pacific-Blue-conjugated anti-CD19 (6D5), FTIC-conjugated anti-IgG1 (A85-1), PE-conjugated anti-IgM (DS-1), PE-conjugated anti-IgM^a^ (DS-1) (BD Biosciences, San Jose, CA). MOG-specific B cells were detected with single-biotin recombinant mouse MOG_tet_ using streptavidin-FITC or –PE. Viability of cells was evaluated by FACS analysis using 7AAD staining (BioLegend, San Diego, CA). For intracellular staining, cells were fixed and permeabilized in 4% paraformaldehyde/0.1% saponin in HEPES-buffered HBSS and stained intracellularly using the following antibodies: FITC-conjugated anti-IgG1 (185-1), PE-conjugated anti-IgM (DS-1). Samples were acquired using a BD LSRFortessa™ X-20 Cell Analyzer. For the iGB cultures CD19^+^CD20^+^CD4^-^ singlets or MOG-tetramer^+^ CD19^+^CD20^+^CD4^-^ singlets were directly sorted into individual wells containing CD40LB feeder cells. Cell-sorting was performed using a FACS Aria III (Becton Dickinson). Analysis was performed using FlowJo (FlowJo, LLC, Ashland, OR) software V10.5.3.

### Induced Germinal Center B Cells Single-B-Cell Culture

CD40LB cells are BALB/c 3T3 fibroblasts stably transfected with both CD40L and BAFF that are routinely used to expand purified B cells in a single-cell culture manner (EasySepTM Mouse B cell Isolation Kit, StemCell, Köln, Germany) (42, 43). On day -1, CD40LB feeder cells were seeded into 96-well plates at 600 cells per well in B cell media: RPMI 1640 medium (Gibco™) supplemented with 10% Gibco™ fetal bovine serum, 5.5 X 10^-5^ M 2-ME, 10 mM Gibco™ HEPES, 1 mM sodium pyruvate, 100 U/mL penicillin, 100 µg/mL streptomycin, and 1 X Gibco™ MEME Nonessential Amino Acid (Thermo Fisher Scientific, Waltham, MA). Next day (day 0), recombinant mouse IL-4 (final 2 ng/mL) and IL-21 (final 10 ng//mL) were added to the cultures, and then single B cells were directly sorted into each well using cell sorters (FACS Aria III, Becton Dickinson). On day 2, 50% (volume) of the culture media was removed from cultures and 100% (volume) of fresh BCM was added to the cultures. From day 3 to day 12 maximum, two-thirds of the culture media was replaced with fresh BCM every day. Depending on the cluster’s quantity and viability, culture supernatants were harvested between 8 and 12 days for ELISA-analyses, B cell clusters harvested for RNA extraction. To establish cell expansion, we FACS sorted 20 polyclonal or MOGtet+ B cells from each studied organ of each mouse in a dedicated well of a CD40LB containing 96 well plate. Cell expansion was analysed after culture by counting viable cells on a Malassez^®^ with blue trypan dead-cell exclusion. The cell expansion was calculated by dividing the total cell number by the 20 cells originally plated by FACS cell sorting.

### Determination of Serum Titers of MOG-Specific Antibodies and of Total IgG1 Antibodies (ELISA)

Serially diluted serum collected from the indicated mice were transferred to 96-well ELISA plates Nunc MaxiSorp^™^ (Thermo Fisher Scientific, Waltham, MA) precoated with rMOGm or purified anti-IgG1 antibodies. After extensive washing, bound Ig was detected by a sandwich consisting of a biotinylated allotype- and isotype-specific anti-mouse IgG1 labelled with streptavidin-HRP (BD Biosciences, San Jose, CA). A colorimetric reaction was performed with an ATBS (2,2’-Azinobis [3-ethylbenzothiazoline-6-sulfonic acid]-diammonium salt) substrate and absorbance was measured at 405 nm.

### RNA Extraction and PCR Analysis for Repertoire Analysis

Total RNA was isolated from B cells clusters after iGB-single culture by TRI Reagent extraction (TRI Reagent^®^ Merck KGaA, Darmstadt, Germany) or the RNAeasy Mini Kit (Qiagen, Courtaboeuf, France), extracted RNA was treated with DNase I (0.1 U/µL, Invitrogen) and cDNA was synthesized using either hexanucleotide or oligo-dT primers and Superscript II Reverse Transcriptase (Invitrogen). We designed and validated primer sets to amplify VDJ gene segments of both the IgG kappa light chain (Igk) and heavy chain (IgHG). To this end, RNA was extracted from iGB cells and reverse-transcribed. Using Igk primer pairs a 500 bp cDNA amplicon was generated encoding the VJ region from either SJL/j or C57Bl/6 mice (**Supplementary Figure S1**). The IgHG primer pair amplified a 400 bp cDNA amplicon from the SJL/j, but not C57Bl6 mice, encoding the IgHG VDJ regions. We next proceeded to determine the BCR rearrangements of the clonal iGB cultures. iGB RNA was extracted from wells with confirmed B cell clusters and reverse-transcribed into cDNA. The variable regions of the IgHG and Igk were amplified by PCR using the above-mentioned primers (**Supplementary Table 1**). PCR products were purified using the QiAquick Gel extraction kit (Qiagen, Courtaboeuf, France) and sequencing confirmed variable parts of murine IgHG and IgHk chains (**Supplementary Figure S1**). PCR conditions used in this study are as follows: IgHG PCR, 94°C for 3 min, followed by 20 cycles of 94°C for 10 s, 67°C for 20 s, 72°C for 20 s, with a progressive determined variation of 0.5°C per cycle;35 cycles of 94°C for 30 s, 57°C for 30 s, 72°C for 1 min; and 72°C for 10 min; and gHk PCR, 94°C for 3 min, followed by 20 cycle of 94°C for 10 s, 68°C for 10 s, 72°C for 20 s with a progressive determined variation of 0.5°C per cycle. VDJ amplimers were gel purified using the QiAquick Gel extraction kit. Sanger sequencing was performed by GENOSCREEN (Lille, France). Sequences obtained for Igk and IgHG chains were submitted to IMGT/V quest and tools (http://www.imgt.org/) to define junction decryptions for each sequences and corresponding amino-acid sequences to determine the different clonotypes.

### Statistics

GraphPad PRISM version 9 (San Diego, CA) was used for statistical analyses and graphs. EAE incidence and mortality was analyzed by Kaplan-Meier plots and statistical significance was calculated using the log rank test. Two-way ANOVA was used to compare the clinical evolution of EAE. Linear regression was used to evaluate correlations. A two-tailed unpaired t-test was used for comparing antibody detection, B cell frequencies and the cumulative disease scores. Levels of statistical significance are graded as follows: NS, Not Significant; *p < 0.05; **p < 0.01; ***p < 0.001; ****p < 0.0001.

## Results

### Spontaneous EAE in TCR^1640^ SJL/j Mice

**Figure 1:**
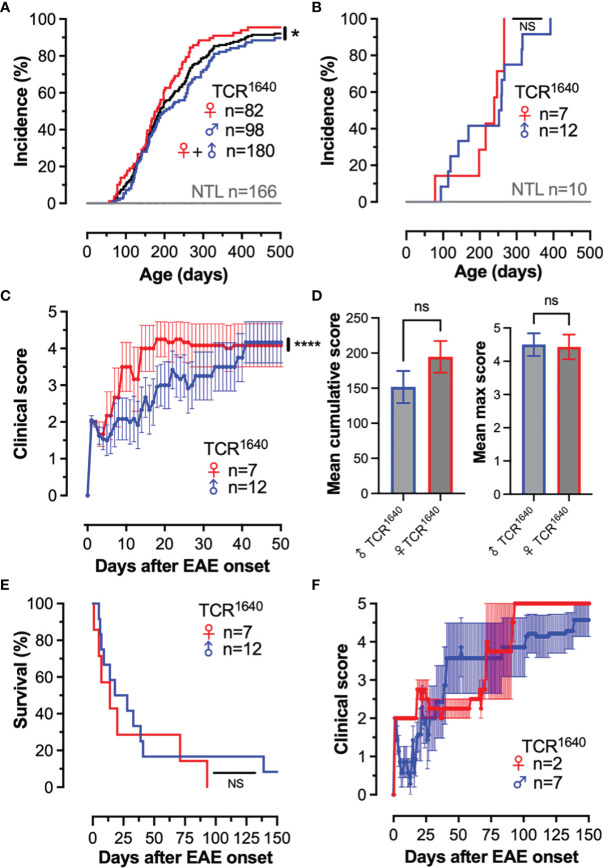
Development of spontaneous progressive EAE in TCR^1640^ mice. **(A)**: Disease incidence over time. EAE onset was monitored twice weekly among male TCR^1640^ (Blue; n = 98) and female TCR^1640^ (Red; n = 82) together with their non-transgenic littermates (Grey; n = 166). Gender independent disease incidence (Black; n = 180). 50% of the TCR^1640^ mice developed EAE after 225 days of age and 80% of mice developed disease after 300 days of age. **(B–F)** Clinical evolution of a dedicated cohort of 19 TCR^1640^ mice and 10 non-transgenic littermates that was monitored daily. **(B)** Disease incidence over time. Male TCR^1640^ (Blue; n = 12) and female TCR^1640^ (Red; n = 7) together with their non-transgenic littermates (Grey; n = 10). **(C)** Clinical disability of female (n = 7) and male (n = 12) TCR^1640^ is presented as the mean EAE score ± SEM for the first 50 days after disease onset. **(D)** Mean cumulative score (left) and mean maximum score (right) for the first 50 days after disease onset. **(E)** Kaplan-Meier curve presenting the probability of survival after disease onset for the male TCR^1640^ (Blue; n = 12) and female TCR^1640^ (Red; n = 7) mice. An acute mortality of 50% is observed for TCR^1640^ mice within 19 days after disease onset. **(F)** The clinical evolution of the remaining 50% of mice surviving beyond 19 days is presented. The mean chronic progressive score +/- SEM is presented for 9 TCR^1640^ mice; male TCR^1640^ (Blue; n = 7) and female TCR^1640^ (Red; n = 2). Log-rank tests were performed for comparisons **(A, B, E)**; 2-way ANOVA **(C)**; Student t-test (**D**, left); Mann Whitney U test (**D**, right). NS, Not Significant; *p < 0.05; ****p < 0.0001.

We established the disease incidence and evolution of the TCR^1640^ mice in our local SPF animal facility. Our colony was rederived by *in vitro* fertilization and the first 2 filial generations were analyzed for spontaneous disease evolution. Animals were scored twice weekly for presence or absence of EAE. As demonstrated in **Figure 1A**, over a period of 500 days 94% of mice spontaneously developed EAE, with an increased incidence for female mice. None of the 166 non-transgenic littermates (NTL) developed disease. In the TCR^1640^ mice earliest EAE onset was observed after 57 days with an average onset for the cohort of 197 ± 87 days. Practically, between the age of 60-500 days the TCR^1640^ mice developed EAE at an average frequency of 1.5% per week.

A dedicated cohort of 19 TCR^1640^ mice and 10 non-transgenic littermates was monitored daily for a period of 150 days (**Figures 1B–F**). None of the NTL developed EAE, while all of the TCR^1640^ littermates spontaneously developed EAE (**Figure 1B**). Most of the TCR^1640^ mice developed a paralytic disease without relapses, which flared more intensely in female mice (**Figure 1C**). No statistically significant differences were observed for the mean cumulative score and mean maximum score between male and female mice (**Figure 1D**). Mortality exceeded 90% and occurred as early as 6 days after disease onset (**Figure 1E**). In half of the diseased TCR^1640^ mice (46.7%) the clinical evolution was acute causing mortality within 19 days (10.9 days ± 5.7) (**Figure 1E**). The other half that survived beyond 19 days developed a chronic progressive disease (**Figure 1F**).

Our results corroborate the susceptibility of the TCR^1640^ mice to spontaneous autoimmune encephalomyelitis. Contrary to the original description (36), our colony develops an acute or chronic disease as opposed to the relapsing-remitting disease originally reported for the TCR^1640^ mice.

**Figure 2:**
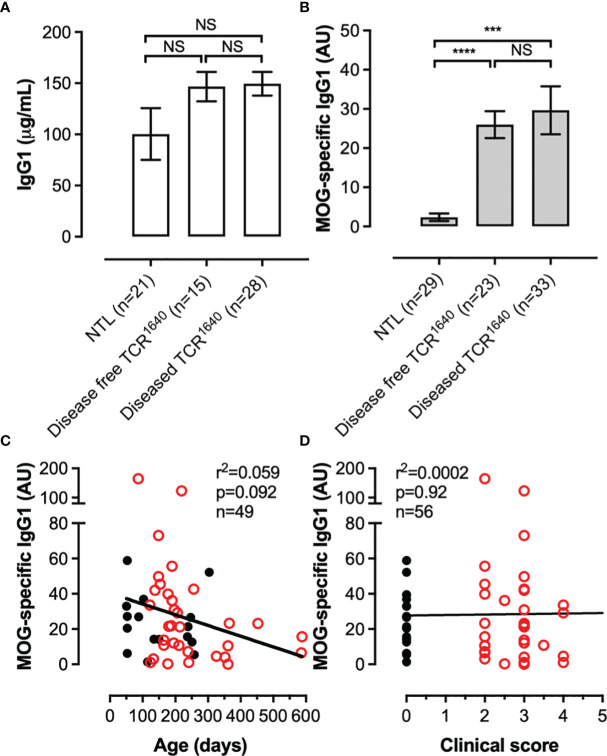
A MOG-specific humoral response develops in TCR1640 mice independent of clinical disease. **(A)** Total IgG1 and **(B)** MOG-specific IgG1 antibodies were detected in the sera of NTL, Disease-free TCR^1640^ and diseased TCR^1640^ mice. Data represents the mean ± SEM of n serum samples. Correlation with **(C)** age or **(D)** clinical intensity of MOG-antibody titres for individual disease-free TCR^1640^ (Black circles, n = 23) and diseased TCR^1640^ mice (Red circles, n = 33). Student t-test **(A, B)**; Fisher exact for linear regressions **(C, D)**. NS, Not Significant; ***p < 0.001; ****p < 0.0001.

We next assessed if the chronic EAE in TCR^1640^ mice mobilizes the humoral response, as has been reported for the initial RR variant (36). The TCR^1640^ expressed on 70% of CD4 T cells is specific for one I-A^s^ restricted epitope of myelin oligodendrocyte glycoprotein (MOG_92-106_) a highly encephalitogenic myelin protein. Pathogenic MOG-antibodies recognize conformational epitopes. To detect their presence in peripheral blood we analyzed their titers by ELISA using recombinant mouse MOG_1-125_. Serum was collected from diseased TCR^1640^ mice during the first five days after onset together with age- and sex-matched non-transgenic littermates and disease-free TCR^1640^ mice (**Figure 2**). Compared to NTL mice, a significant increase in anti-MOG antibodies was detected in the sera from TCR^1640^ mice, which is coherent with the development of a MOG-specific humoral response in diseased TCR^1640^ mice (**Figure 2B**). Unexpectedly, the disease free TCR^1640^ mice similarly developed a MOG-specific IgG1 response that was significantly increased compared to NTL mice and similar to that detected in diseased TCR^1640^ mice (**Figure 2B**). Total IgG1 titers did not significantly differ between sera from healthy and disease TCR^1640^ mice and NTL mice (**Figure 2A**).

We next correlated the MOG-antibody titers with age and disease severity in order to assess if these factors influence the MOG humoral response. As demonstrated in **Figures 2C, D** neither disease severity in diseased TCR^1640^ mice or age of disease-free and diseased TCR^1640^ mice significantly influenced MOG-antibody titers.

**Figure 3:**
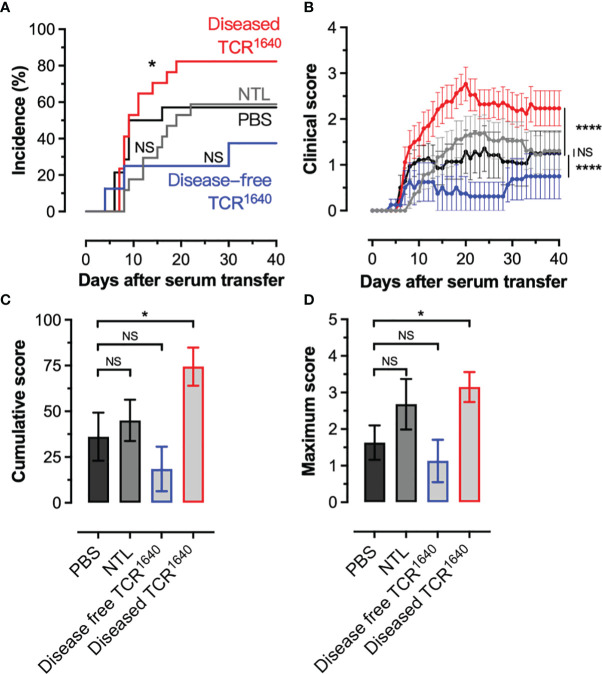
Pathogenicity of MOG-antibodies by serum transfer into EAE prone recipients. **(A, B)** Mild EAE develops in 2D2 MOG-TCR Tg mice after 2 injections (Days 0, 2) of *pertussis* toxin (Incidence 50%). These PT treated 2D2 mice received PBS (n=14) or serum from disease-free TCR^1640^ mice (n = 8), diseased TCR^1640^ mice (n = 17) or NTL mice (n = 18) 2 days after the first PT injection. **(A)** Incidence of EAE over time. **(B)** Mean clinical disability ± SEM presented over time (days after serum transfer). Log-rank test **(A)**, 2-way ANOVA **(B)**, student t-test **(C)**, Mann-Whitney U test **(D)** was used to compare the clinical evolution of EAE. The data was pooled from 4 individual experiments in which sera from single donor mice were transferred into individual 2D2 recipients. NS, Not Significant; *p < 0.05; ****p < 0.0001.

To assess the pathogenicity of the humoral response we transferred individual sera from NTL and TCR^1640^ mice into EAE-prone 2D2 TCR transgenic mice. These mice carry a MOG_35-55_ specific transgenic TCR on the C57BL/6 background, but do not develop a MOG-specific B cell response/anti-MOG antibodies (40). Spontaneous EAE development is observed in 1% of our 2D2 colony (data not shown), however, *i.v.* injections of pertussis toxin at a 2-day interval allows for EAE development in 50% of 2D2 mice (**Figure 3A**). When transferring 50 μg of the MOG-specific IgG1 mAb 8-18C5 EAE incidence is raised to 100% causing severe paralytic disease (**Supplementary Figure S2**). The transfer of sera from NTL failed to significantly alter disease incidence and EAE severity compared to PBS controls (**Figures 3A, B**). No difference was observed for the cumulative disease score or the maximum disease score (**Figures 3C, D**). Transfer of sera from disease-free TCR^1640^ mice resulted in disease with reduced severity compared to the PBS controls that reached statistical significance with the 2-way ANOVA (**Figure 3B**). No statistically significant variance was observed for disease incidence, cumulative score, or maximum score between sera from the disease-free TCR^1640^ mice and PBS controls (**Figures 3A, C, D**). By contrast, sera from diseased TCR^1640^ mice induced EAE in 82% 2D2 recipients (**Figure 3A**) and significantly increased the cumulative EAE score relative to the NTL-serum and PBS injected groups (**Figure 3B**). Both the cumulative disease score and maximum disease score increased significantly after transfer of sera from diseased TCR^1640^ mice (**Figures 3C, D**). These findings demonstrate that sera containing MOG-specific antibodies of adult TCR^1640^ mice exacerbate disease in EAE-prone recipients only when derived from diseased TCR^1640^ donor mice.

### Tracking MOG-Specific B Cells in the Secondary Lymphoid Organs and Central Nervous System of TCR^1640^ Mice

**Figure 4:**
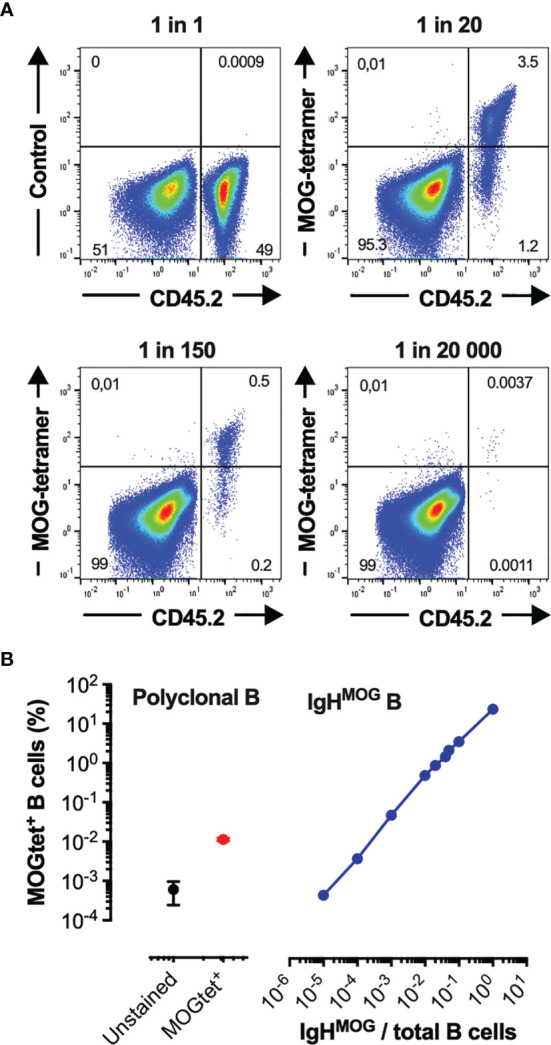
Validation and sensitivity of the recombinant MOG_1-125_-tetramer. **(A, B)** Purified CD45.2^+/+^ B cells from homozygous C57Bl/6 IgH^MOG^ mice were mixed *in vitro* with purified B cells from congenic CD45.1^+/+^ mice at the indicated ratios. **(A)** A representative flow cytometry profile demonstrating the capacity of Streptavidin-FITC MOG_1-125_-tetramers to identify IgH^MOG^ B cells among polyclonal CD45.1^+/+^ B cells (Top right, bottom), the Streptavidin-FITC MOG_1-125_-tetramers were omitted in the control samples (Top left) **(B)** Sensitivity of the Streptavidin-FITC MOG_1-125_-tetramers in detecting the precursor frequency of the IgH^MOG^ B cells. Left panel, MOG-tet^+^ staining of CD45.1^+/+^ polyclonal B cells (red circle), and the control staining without the MOG_tet_ (black circle). Right panel, MOG-tet^+^ staining off CD45.2^+/+^ IgH^MOG^ B cells serially diluted among CD45.1^+/+^ polyclonal B cells (blue line). The mean +/- SEM is presented from 2 pooled experiments.

**Figure 5:**
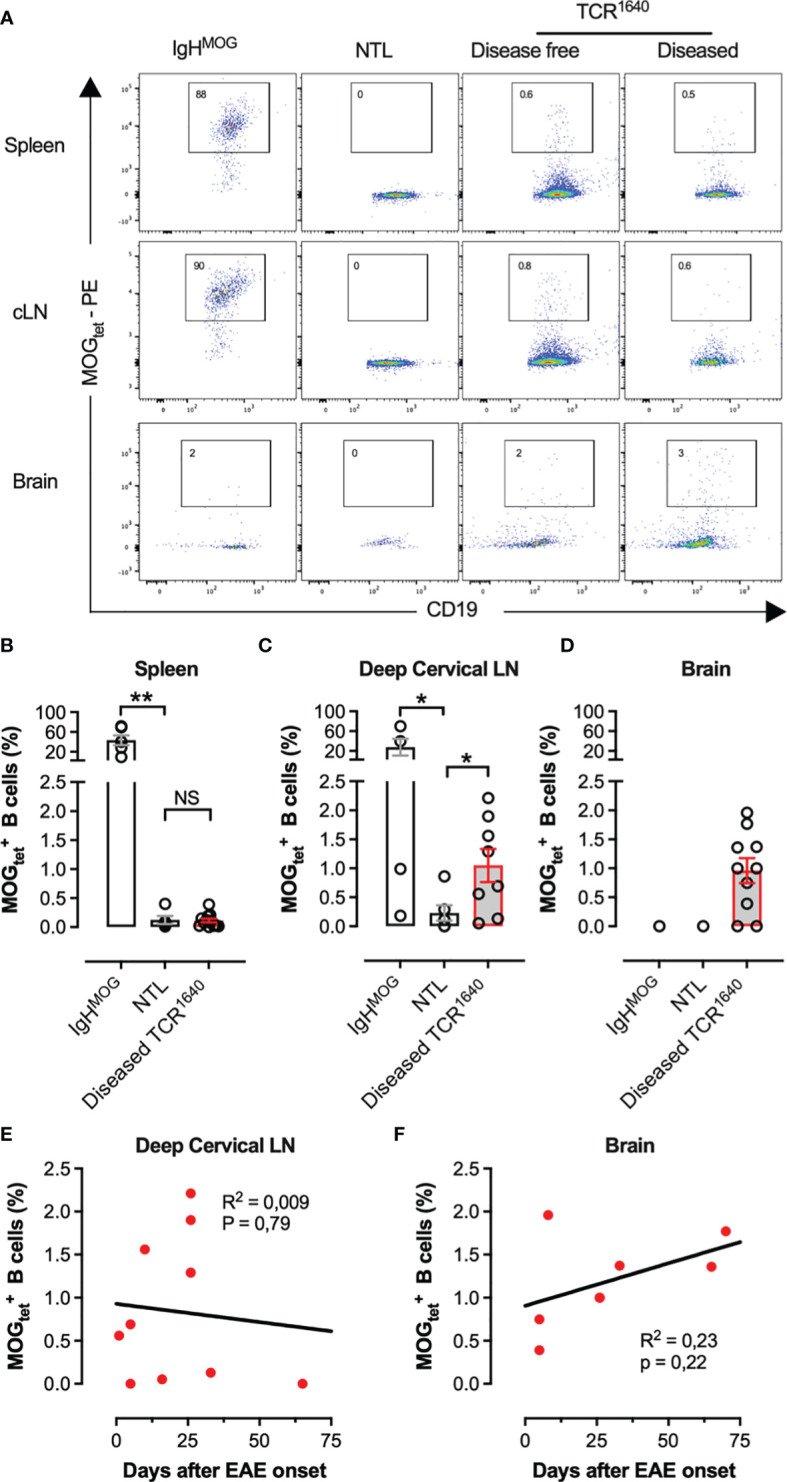
Frequency and localisation of ${\text{MOG}}_{\text{tet}}^{+}$ B cells in TCR^1640^ mice; **(A)** A representative flow cytometry profile gated on viable CD4^neg^ CD20^+^CD19^+^ B cells. MOG_1-125_-tetramer staining of single cell populations from the spleen (top row), cervical lymph nodes (middle row), and percoll purified mononuclear cells from the brain (Bottom row) are presented from IgH^MOG^ mice (Left column), NTL (center left column), disease-free TCR^1640^ (center right column) and diseased TCR^1640^ mice (right column). Data representative of 3 independent experiments. **(B)** Precursor frequency of ${\text{MOG}}_{\text{test}}^{+}$ B cells in the spleen (IgH^MOG^ n = 6; diseased TCR^1640^ n = 12; NTL n = 5), **(C)**, cervical lymph nodes (IgH^MOG^ n = 4; diseased TCR^1640^ n = 8; NTL n = 6), and **(D)** brain (IgHMOG n = 2; diseased TCR^1640^ n = 10; NTL n = 3). The mean frequency of n samples +/- SEM is presented. **(E, F)** Correlation between the frequency of ${\text{MOG}}_{\text{test}}^{+}$ B cells and the delay after EAE onset in deep cervical lymph node **(E)** or in brain **(F)** in diseased TCR^1640^ mice. Student t-test **(B, C)**; Fisher exact for linear regressions **(E, F)**. NS, Not Significant; *p < 0.05; **p < 0.01.

To identify and track MOG-specific B cells we produced single-biotin avitagged recombinant mouse MOG_1-125_ which was tetramerized using fluorochrome labelled streptavidin (MOGtet). To assess the sensitivity of the MOG_tet_ we serially diluted MOG-specific B cells from CD45.2^+/+^ IgH^MOG^ mice among CD45.1^+/+^ B cells from wild-type C57BL/6 mice (**Figure 4**). The MOG tetramer specifically stained the congenic IgH^MOG^ B cells, with only low frequency labelling among the polyclonal B cells (**Figure 4A**). The IgH^MOG^ MOG-specific B cells could be detected at a minimal frequency of approximately 1 in 10^4^ B cells (**Figure 4B**). Next, we performed *ex vivo* analysis of the B cell compartment of NTL and TCR^1640^ mice to enumerate the MOG-specific B cell response. On average 73+/-10% (SEM) of B cells were positive in the MOGtet staining in IgH^MOG^ mice which were included as a positive control (**Figures 5A, B**). By focusing primarily on brain-infiltrating MOG-specific B cells, we chose to preserve all TCR^1640^ mice until the development of clinical EAE. The 2 disease-free mice were excluded from the analysis in **Figures 5B–D** because of the low number of mice for statistics. In adult NTL the frequencies of ${\text{MOG}}_{\text{tet}}^{+}$ B cells remained below 0.1% in the spleen and below 0.2% in the brain-draining cervical lymph nodes (cLN) (**Figure 5B**). Upon development of disease the TCR^1640^ mice demonstrated an increase in MOGtet^+^ B cells reaching an average of 1.0+/-0.3% in the cLN (**Figure 5C**). A similar presence of ${\text{MOG}}_{\text{tet}}^{+}$ B cells was detected among the mononuclear cells isolated from pooled brains of diseased TCR^1640^ mice, which averaged at 1.0+/-0.2 (**Figure 5D**). These data suggest that in diseased TCR^1640^ mice the MOG-specific B cells reside predominantly in the cLN and brain. We next correlated MOG-specific B cell frequencies with the time after EAE onset to assess any temporality in MOG-specific B cells during EAE progression. In the inflamed brain the proportion of ${\text{MOG}}_{\text{tet}}^{+}$ B cells remained relatively stable over time while the precursor frequency of ${\text{MOG}}_{\text{tet}}^{+}$ B cells in the cervical lymph nodes was more varied (**Figures 5E, F**). This dataset is coherent with the activation of MOG-specific B cells in the brain-draining cLN prior to infiltration of the inflamed brain.

### Clonality of the MOG-Specific B Cell Response

Adopting a culture system for expansion and differentiation of B cells developed by Kitamura and colleagues, we established a clonal B cell expansion protocol using 3T3 fibroblasts stably transfected with both CD40L and BAFF (CD40LB cells) supplemented with IL-4 and IL-21 (42). This clonal amplification strategy multiplies the genetic material required to sequence the BCR rearrangement of paired heavy and light chains. In parallel, antigen-specificity is determined by ELISA using the supernatants after clonal B cell expansion.

**Figure 6:**
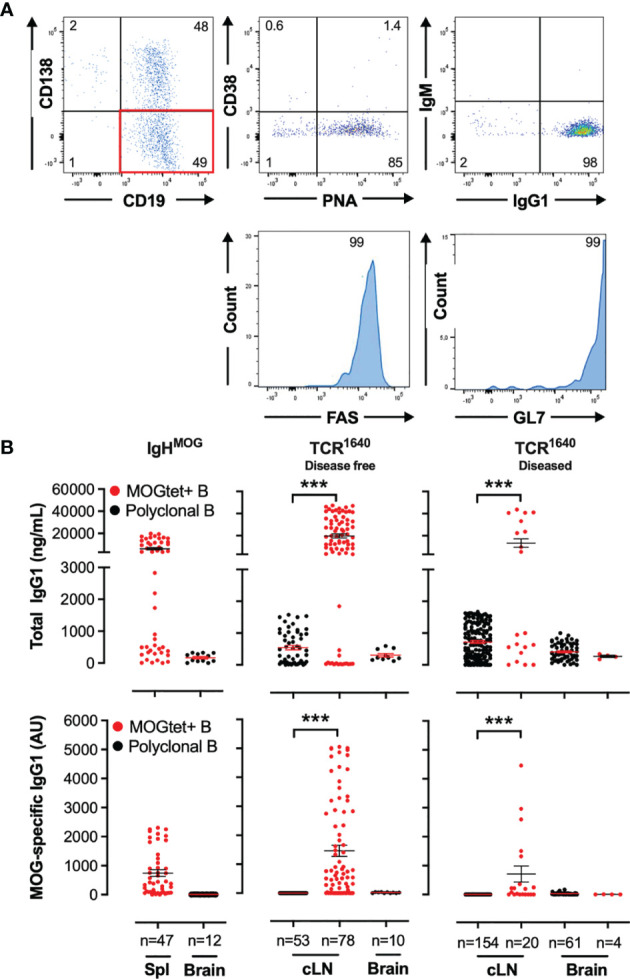
Identification of MOG-specific B cell clones by measuring total IgG1 and MOG-specific IgG1 antibody titres in the supernatants from single-cell iGB cultures. **(A)** Differentiation of clonal B cells into CD138^+^ plasma cells and CD38^-^ GL7^+^FAS^+^PNA^+^ germinal centre B cells. iGB cultures of single B cells isolated from the cervical lymph nodes and brain of diseased TCR^1640^ mice were analysed by flow cytometry after 8 days of culture. A representative profile of viable CD19^+^ B cells demonstrate presence of CD138 plasma cells (Top row, left plot), and gated on CD19^+^CD138^-^ B cells (Red quadrant) the expression of CD38 and PNA (top middle, intracellular IgG1 and IgM (top right), and histograms for FAS (bottom left) and GL7 (bottom right). **(B)** Total IgG1 (top row) and anti-MOG IgG1 (bottom row) antibodies were detected in supernatants from spleen, cervical lymph nodes, and brain-derived single B cell iGB cultures from IgH^MOG^ (Left column), disease-free TCR^1640^ (middle column) and diseased TCR^1640^ mice (right column). Clonal B cells were isolated from the polyclonal CD19^+^ B cell compartment (black circles) or from the CD19^+^ ${\text{MOG}}_{\text{test}}^{+}$ B cell response (red circles). The individual antibody concentration of n clonal iGB cultures is presented together with the Mean +/- SEM. Data represents the pooled results of 3 independent experiments and was analysed with a t-test. ***p < 0.001.

**Supplementary Figure S3** and **Figure 6** show the efficacy of expansion of single B cells isolated from BALB/c spleen and from brain infiltrating single B cells of diseased TCR^1640^ mice, respectively, in the induced germinal B cell (iGB) culture. B cell expansion was accompanied by differentiation. *In vitro* flow cytometry analysis of the B cell clusters demonstrated that half of the B cells had differentiated into CD138-positive plasmablasts while the phenotype of the other half showed a germinal center B cell phenotype with limited CD38 expression and strong induction of FAS, GL7 and Peanut agglutinin (**Figure 6A**). In response to the added cytokines IL-4 and IL-21, isotype switching from IgM to IgG1 was observed for 98% of B cells (**Figure 6A**).

The efficacy of expanding single B cells in iGB cultures was similar for B cells isolated from the cLN of disease-free and diseased TCR^1640^ mice. B cell expansion was observed for 46% and 39% of clones with an average fold amplification of 6300 and 4588 for disease-free versus diseased TCR^1640^, respectively (**Supplementary Figure S4**). Sorting MOG_tet_-positive B cells from the cLN of disease-free and diseased TCR^1640^ mice permitted the expansion of 40% and 39% of clones with a 5167 and 3233 fold-amplification, respectively (**Supplementary Figure S4**). This demonstrates the efficacy of single-cell iGB cultures derived from the secondary lymphoid organs. The efficacy proved more modest for effector B cells isolated from the brain. Single B cell cultures of B cells from disease-free and diseased TCR^1640^ mice induced expansion in only 3.6% and 13.1% of cultures with a fold-amplification of 1217 and 2686 (**Supplementary Figure S4**). Isolating ${\text{MOG}}_{\text{tet}}^{+}$ B cells from the brain of diseased TCR^1640^ mice proved most challenging as only 4% of single-cell cultures showed evidence of abortive proliferation by B cell clusters evolving to rapid cell death (**Supplementary Figure S4**).

To establish specificity of the B cell clones for MOG we collected the culture supernatants of proliferating cultures and determined the concentration of MOG-specific antibodies by ELISA (**Figure 6B**). MOG-specific antibody production could not be detected among the 47 splenic iGB clones derived from NTL mice (data not shown), which is coherent with the low precursor frequency of MOG-specific B cells in the polyclonal repertoire of wild-type mice. For the single cell iGB cultures from the TCR^1640^ mice sorting antigen-specific B cells *via* MOGtet-staining significantly enhanced the efficacy of isolating MOG-specific B cell clones (**Figure 6B**, bottom row). ${\text{MOG}}_{\text{tet}}^{+}$ iGB cultures also resulted in higher IgG1 titers compared to the polyclonal iGB cultures. In the ${\text{MOG}}_{\text{tet}}^{+}$ iGB cultures from IgH^MOG^ mice all IgG1 antibody producing cultures were MOG-specific (**Figure 6B**). iGB cultures from MOGtet^+^ B cells derived from TCR^1640^ mice induced IgG1 antibody production of which 69% were MOG specific in disease-free mice and 50% were MOG-specific in diseased mice (**Figure 6B**).

The abortive proliferation of brain-derived ${\text{MOG}}_{\text{tet}}^{+}$ iGB cultures probably underlies the absence of detectable MOG-specific antibodies in the culture supernatants. Taken together these data demonstrate the efficacy of iGB cultures in expanding polyclonal or tetramer-binding B cells from secondary lymphoïd organs. The iGB cultures proved less efficient in expanding autoantigen-specific B cell clones that were isolated from the inflamed target tissue.

### Repertoire Analysis

Few studies have addressed the B cell repertoire in SJL/j mice. Here, a total of 380 iGB cell clusters were analysed. From disease-free TCR^1640^ mice individual polyclonal B cells from the cLN (n=53) and brain (n=10) as well as ${\text{MOG}}_{\text{tet}}^{+}$ B cell clones from the cLN (n=78) were sequenced. From diseased TCR^1640^ mice individual polyclonal B cells from the cLN (n=154) and brain (n=61) as well as ${\text{MOG}}_{\text{tet}}^{+}$ B cell clones from the cLN (n=20) and brain (n=5) were sequenced. Only 11 IgHG and 21 Igk sequences were obtained (**Tables 1**, **2**) and their VDJ rearrangements were analysed using the IMGT website (http://www.imgt.org). As presented in **Table 1**, 11 unrelated IgHG sequences were obtained providing no evidence of *in vivo* clonal expansion. By contrast, the Igk light chains did provide evidence of selected CDR3 sequences. **Table 2** presents the 21 obtained Igk sequences that were segregated based on MOG specificity. The CDR3 encoding junctional sequences revealed 3 consensus sequences of which 2 are confirmed to be derived from MOG-specific B cell clones. The “CQQXSSYPXTF” sequence was deduced from 13 iGB clones that were derived from diseased (n=11), but also from the cLN of non-diseased (n=2) TCR^1640^ mice. MOG-specificity was confirmed for one clone isolated from brain (n=4) and one clone derived from the cLN (n=7) of diseased TCR^1640^ mice. The majority of iGB clones produced total IgG titers below 1 μg/ml, a concentration insufficient to reliably detect MOG antibodies (ND). A second consensus light chain sequence derived from MOG-specific iGB clones was identified as “CQQHNEXPWTF”. This consensus junctional sequence was obtained from clonal B cells isolated from the brain and cLN of diseased TCR^1640^ mice. A third consensual junctional sequence was identified as “CQXGVVTHPX” on kappa light chains derived from the cLN of non-diseased TCR^1640^ mice and was not associated to MOG-reactivity. 2 heavy and light chain pairs were identified. The kappa light-chain V15-103*01-J1*02 with the consensual junctional sequence CQQXSSYPXTF was paired with the heavy chain V5-17*01, D1-1*01, J3*01, CXXXYXYGSTAWFAXW. The second BCR comprised a kappa light-chain V16-104*01, J1*01with the consensual junctional sequence CQQ HNE XPW TF was paired with the heavy chain V1-18*01, D2-3*01, J4*01, XQEGXGISMLWTT. The consensual amino-acid sequences of the junctional region were submitted to the protein BLAST tool from NCBI with mus musculus (taxid:10090) as targeted organism (http://blast.ncbi.nlm.gov/Blast.cgi?PAGE=Proteins). This short consensual amino-acid sequence for Igk probed 97 different hits representing for the large majority of other light chain sequences. The distance tree of this analysis (**Supplementary Figure S4**) corroborated the relevance of this consensual amino-acid sequence to rodents and especially mus musculus as part of the Igk region sequences (Fast Minimum Evolution, 0.90 of max sequence difference, distance of Grishin for proteins). These results indicate that despite the limited efficacy shared light chain rearrangements could be identified in the cLN and brain of the TCR^1640^ mice suggestive of clonal expansion.

**Table 1:** VDJ rearrangements and junctional sequence of the IgG heavy chain of clonal iGB B cells.

MOGtet^+^	MOGAbs	EAE	Organ	Clonotype	V-geneallele	D-geneallele	J-geneallele	AA junction
**-**	**+**	3.5	cLN	6	V1-80*01	D1-1*01	J2*03	CAR RGY XXX SXY XXY X
**-**	ND	3	cLN	1	V1-18*01 (a)	D2-3*01	J4*01	XQE GXG ISM LWT T
**-**	ND	3	cLN	3	V5-17*01 (b)	D1-1*01	J3*01	CXX XYX YGS TAW FAX W
**-**	ND	3	cLN	10	V1-50*01	D1-1*01	J3*01	CAR GGY GSS PAW FAX X
**-**	ND	3.5	cLN	2	V1-42*01	D3-1*01	J*03	XAR RXR SXW XXX X
**-**	ND	3	cLN	4	V1-18*01	D2-3*01	J2*03	XAR WGG GFX YX
**-**	ND	3	cLN	5	V1-69	D1-1*01	J4*01	CAR RGX XSX SXX XXS
**-**	ND	3	cLN	7	V1-18*01	D2-1*01	J3*01	CAR VNL AWF XYW
**-**	ND	0	cLN	8	V1-39*01	D4-1*01	J2	CAR RLG REX XXX X
**-**	ND	3	cLN	9	V1-82*01	–	J2	CAR GXY W
**-**	ND	3	cLN	11	V1-53*01	D4-1*01	J2	CAR ERX GXX YX
						Consensus sequence		CAR XXX XXX

The first two columns indicate MOG-specificity determined as MOG-tetramer binding during cell-isolation (column 1) or MOG-antibody production determined by ELISA in the iGB culture supernatant (column 2). Columns 3 and 4 refer to the EAE clinical score at the day of sacrifice and the organ from which the B cell clones were isolated. BCR rearrangement is presented as the individual V-D-J gene alleles and the junctional sequence of the hypervariable region. ND (Not detected). (a) and (b) refer to the pair (a) or (b) of IgG heavy and kappa chains from the same iGB culture well (see **Table 2**).The * refers to the immunoglobulin nomenclature which defines the use of an asterix to refer to different allele polymorphisms. + means positive; - means negative.

**Table 2:** VJ rearrangements and junctional sequence of the kappa light chain of clonal iGB B cells.

**MOGtet^+^**	**MOGAbs**	**EAE**	**Organ**	**Clonotype**	**V-geneallele**	**D-geneallele**	**J-geneallele**	**AA junction**
**-**	**+**	3	Brain	2	V2-109*01	–	J4*01	CXQ NXE XPW TF
**+**	**+**	3	cLN	3	V4-57-1*01	–	J1*01	CQQ HNE XXW TX
**-**	ND	3	cLN	4	V16-104*01 (a)	–	J1*01	CQQ HNE XXW XX
						Consensus sequence		CQQ HNE XPW TF
								
**-**	ND	3	Brain	3	V1-133*01	-	J1*01	CVQ GTH FPX TF
**-**	ND	3	cLN	6	V4-55*01	-	J2*01	CQQ XSS YPP T
**-**	-	3	cLN	5	V4-55*01	-	J5*01	CQQ WSS YPL TX
**-**	ND	3	cLN	13	V4-55*01	-	J5*01	XQQ WDS YPP TF
**-**	ND	3	cLN	5	V4-57*01	-	J5*01	CQQ RSS YPX TX
**-**	**+**	3	Brain	4	V4-61*01	-	J1*01	CQQ YXS YPR TX
**-**	ND	3	cLN	4	V4-92*01	-	J5*01	CQQ GSS SPL TX
**+**	**+**	3	cLN	5	V8-24*01	-	J4*01	CQQ XSG XPX TX
**-**	ND	0	cLN	1	V8-24*01	-	J2*01	CQQ HYX TPY TX
**-**	-	0	cLN	3	V8-19*01	-	J5*01	CQN DXS XPX TX
**-**	ND	3	Brain	2	V8-28*01	-	J4*01	CXQ DHS YPF TX
**-**	ND	3	Brain	1	V8-30*01	-	J2*01	CQQ YYS YPY TF
**-**	ND	3	cLN	12	V15-103*01 (b)	-	J1*02	XXQ GXS XXX TX
						Consensus sequence		CQQ XSS YPX TF
**-**	ND	0	cLN	7	V4-81*01	-	J5*01	XQX GVV THP X
**-**	ND	0	cLN	8	V6-15*01	-	J1*01	CQX XVV THP X
						Consensus sequence		CQX GVV THP X
**-**	ND	3	cLN	9	V3-9*01	-	J3*01	XCK VGR FRG RX
**-**	ND	0	cLN	10	V5-48*01	-	J5*01	CQQ SNX GQP R
**-**	ND	3	cLN	11	V19-93*01	-	J1*01	CLQ YDN LXT X
								
						Consensus sequence		CXQ XXX XXX RX

The first two columns indicate presence (+) of MOG-specificity determined as MOG-tetramer binding during cell-isolation or MOG-antibody production in the iGB culture supernatant.

Columns 3 and 4 refer to the EAE clinical score at the day of sacrifice and the organ from which the B cell clones were isolated. BCR rearrangement is presented as the individual V-J gene alleles and the junctional sequence of the hypervariable region. The top two consensus sequences (or clonotypes), in white and pink chadings, are associated with MOG-antibody producing and MOGtet+ B cell clones. The third consensus sequence (or clonotype), in orange shading, was identified among cLN B cells of disease free TCR1640 mice. The last group of remaining sequences provided no linear homology (Grey). ND (Not detected). (a) and (b) refer to the pair (a) or (b) of IgG heavy and kappa chains from the same iGB culture well (see **Table 1**).The * refers to the immunoglobulin nomenclature which defines the use of an asterix to refer to different allele polymorphisms.

## Discussion

The environment critically influences autoimmune disease (44). Female non-obese diabetic mice spontaneously develop type 1 diabetes in SPF animal facilities while they remain healthy in conventional environments (45). This extends to spontaneous animal models of MS-like disease that have emerged among MOG-specific TCR transgenic mice. On the C57Bl/6 background, the I-A^b^ : MOG_35-55_ specific 2D2 TCR transgenic mice were originally ascribed to develop optic neuritis (40), but may develop EAE with an incidence of up to 18% (46, 47), and in our facility the 2D2 colony developed EAE with an incidence of 1% (Data not shown, 2D2 = 199; wild type littermates n=203; monitoring of 250 days). The EAE susceptibility of the TCR^1640^ SJL/j colony housed in our facility was preserved as indicated by the 94% EAE incidence at the age of 500 days. Unlike the originally reported relapsing-remitting disease (36), our TCR^1640^ colony spontaneously developed a chronic progressive EAE that proved moribund for half of the mice within 3 weeks of onset. Clinically, disease was delayed with onset at the age of 225 days for 50% of TCR^1640^ mice, as opposed to an age of 110 days for the original TCR^1640^ colony. A gender difference was observed with disease flaring more quickly in females. Our study thus reiterates the environmental influence of EOPS animal facilities in spontaneous EAE in genetically identical mouse strains.

Within the context of spontaneous EAE, this study assessed the mobilization of MOG-specific B cells from the polyclonal B cell repertoire of TCR^1640^ mice. To this end we produced recombinant MOG tetramers to trace the autoantigen-specific B cell response in the TCR^1640^ mice. In concordance with previous studies, ${\text{MOG}}_{\text{tet}}^{+}$ B cells accumulated in the brain-draining cervical lymph nodes and the brain of TCR^1640^ mice that had spontaneously developed EAE. The specificity of the MOG_tet_ was validated using serially diluted IgH^MOG^ B cells among congenic polyclonal B cells indicating that a clonal frequency of high affinity B cells at 1 in 10^3^-10^4^, was detectable (**Figure 4**). We confirmed the specificity of the MOG_tet_ for polyclonal B cells using the clonal ${\text{MOG}}_{\text{tet}}^{+}$ iGB cultures which demonstrated that all IgG1 antibody producing ${\text{MOG}}_{\text{tet}}^{+}$ clones were MOG-specific (**Figures 5**, **6**). The mobilization of the ${\text{MOG}}_{\text{tet}}^{+}$ B cell response precedes clinical EAE. In disease-free TCR^1640^ mice MOG-specific B cells could be detected and their iGB cultures demonstrated the production of MOG-specific antibodies by all IgG1 producing clones (**Figures 5**, **6**). This mobilization of the MOG-specific humoral response could be observed as early as 7 weeks by the detection of circulating MOG-specific antibodies in TCR^1640^ sera of disease-free mice (**Figure 2**). The absence of correlation between MOG-antibody titers and spontaneous EAE onset is undoubtedly mediated by the difficulty for antibodies to penetrate the brain parenchyma under physiological conditions. Passive models of MOG-antibody mediated EAE (notably demonstrated with the mouse IgG1 monoclonal antibody 8-18C5) require moderate neuroinflammation and blood-brain-barrier permeability in order to mediate CNS demyelination (48, 49). Our results, using the adoptive serum transfers to EAE-prone 2D2 recipient mice indicate that factors intrinsic to the immune response might influence pathogenicity. Indeed, only the sera from diseased TCR^1640^ mice aggravated EAE relative to the 2D2 recipients receiving PBS. Sera from the disease-free TCR^1640^ mice failed to do so. While other soluble factors such as cytokines or complement may contribute to this difference, antibody intrinsic factors such as isotype, Fc-glycosylation or affinity maturation by clonal selection or somatic hypermutation are likely implicated.

To study the repertoire, we opted for a focused approach combining MOG-tetramers to isolate single B cells and iGB cultures to expand these clones *in vitro*. This approach would have the advantage of identifying the paired heavy and light chains sequences of the MOG-specific B cell responses. The iGB culture of bulk splenic B cells from naive BALB/c or NTL SJL/j mice, and the single cell culture of total/non-antigen-selected) splenic B cells from naive BALB/c or NTL SJL/j mice were highly efficient. Considering single-cell culture of naïve splenic B cells, 70% of BALB/c B cell cultures expanded with a 10^4^ fold expansion, corroborating previous reports (42). The efficacy of single-cell iGB cultures is known to be reduced in terms of proportion of clones proliferating as well as their fold expansion, with reductions from 60% for splenic B cells from naïve WT mice to 23% for germinal center B cells from immunized mice (50). For the cervical lymph nodes, we observed an efficacy range between 38-46% for single-cell cultures of polyclonal B cells or ${\text{MOG}}_{\text{tet}}^{+}$ B cells from healthy and diseased TCR^1640^ mice. iGB cultures from brain-infiltrating B cells demonstrated low efficacy dropping to only 3.6-13% for polyclonal B cells, and 0-4% for ${\text{MOG}}_{\text{tet}}^{+}$ B cells. CNS infiltrating lymphocytes are effector cells prone to undergo activation induced cell death upon *in vitro* reactivation (50). The CD154 expressing, BAFF producing 3T3 fibroblasts were created for the *in vitro* differentiation of mature B cells into germinal center B cells. When applied to tissue infiltrating effector cells their stimulatory capacity might well be in excess.

Despite the constraints in amplifying B cells from the inflamed target tissue we did pursue the sequencing of the expanded iGB clones to identify potential BCR rearrangements associated with MOG-driven disease. Our results revealed shared light chain rearrangements among B cell clones from TCR^1640^ mice suggestive of clonal expansion. Nevertheless, single-cell transcriptomic approaches on non-expanded *ex-vivo* B cells would be required to study the clonality and somatic hypermutations of the antigen-driven B cell response in the TCR^1640^ mice.

## Conclusion

Our results reiterate that spontaneous EAE in genetically predisposed animals is influenced by the environment under which they are housed. We confirmed that the autoimmune demyelinating humoral response matures in the brain-draining lymph nodes. This process is initiated prior to disease onset, as MOG-antibodies could be detected as early as 7 weeks of age. Sera containing MOG-antibodies proved pathogenic once TCR^1640^ mice had developed disease. The expanded MOG-specific B cell response demonstrated clonal selection as consensus sequences for the junctional region of MOG-reactive kappa light-chains could be identified. Our study indicates that clonal selection and brain infiltration of the MOG-specific humoral response are implicated in a spontaneous model of autoimmune demyelination.

## Data Availability Statement

The datasets presented in this study can be found in online repositories. The names of the repository/repositories and accession number(s) can be found in the article/**Supplementary Material**.

## Ethics Statement

All experimental protocols were approved by the local ethics committee and and the Ministère de l’Enseignement Supérieur de la Recherche et de l’Environnement (5157-2016111011562655) in compliance with European Union guidelines.

## Author Contributions

Conceptualization, AP, LM, HZ. Methodology, MF, NJ, LM, HZ. Investigation, FS, LD, FL, TG, JB, MB, NJ. Data curation, FS, LD, FL, NJ, LM, HZ. Writing, LM, HZ, with input from all authors. Visualization, FS, LD, FL, TG, LM, HZ. Supervision, L.M, HZ. Funding acquisition, AP, LM, HZ. LM and HZ have full access to all the data in the study and take responsibility for the integrity of the data and the accuracy of the performed analyses.

## Funding

FS received a scholarship from Institut Roche. The funder was not involved in the study design, collection, analysis, interpretation of data, the writing of this article or the decision to submit it for publication. LD and JB received research scholarship from the Univ of Lille, Departments of Pharmacy or Medicine, respectively. HZ and AP were supported by ARSEP (call for proposal 2014 and 2016). AP was supported by the Emmy Noether Program of the German Research Foundation DFG (PE 2681/1-1). LM is supported by the Institut National de la Santé et de la Recherche médicale (INSERM), the University of Lille, and grants from the Agence Nationale de la Recherche “sugars-in-MS” (ANR-17-CE15-0028), The French MS society (ARSEP, Fondation pour L'aide à la recherche sur la Sclérose en plaques), and the Haut-de-France région.

## Conflict of Interest

The authors declare that the research was conducted in the absence of any commercial or financial relationships that could be construed as a potential conflict of interest.

## Publisher’s Note

All claims expressed in this article are solely those of the authors and do not necessarily represent those of their affiliated organizations, or those of the publisher, the editors and the reviewers. Any product that may be evaluated in this article, or claim that may be made by its manufacturer, is not guaranteed or endorsed by the publisher.

## Supplementary Material

Supplementary Figure 1 Amplification of the antibody variable region. Validation of the primer pairs presented in **Table 1**. cDNA was obtained from purified B cells/iGB cells of SJL/J and C57BL/6 mice. Amplified PCR products migrated to expected molecular mass size (close to 500 kDa for IgHG and close to 400 kDa for Igk). Sequencing of the purified amplicon for SJL/J mice confirmed the amplification of the hypervariable regions of murine IgHG and Igk chains (data not shown).

Supplementary Figure 2 Pathogenicity of monoclonal 8-18C5 IgG1 MOG-specific antibodies by adoptive transfer into EAE-prone recipients. Mild EAE develops in 2D2 MOG-TCR Tg mice after 2 injections (Days 0, 2) of *pertussis* toxin (Incidence 50%). These PT treated 2D2 mice received PBS (n = 4) or 50 μg of 8-18C5 (n = 4), or 50 μl of NTL serum (n=7) 2 days after the first PT injection. Mean clinical disability ± SEM is presented over time (days after serum transfer). 2-way ANOVA.

Supplementary Figure 3 Differentiation of clonal B cells into CD138^+^ plasma cells and CD38^-^ GL7^+^FAS^+^PNA^+^ germinal centre B cells. **(A)** iGB cultures of single B cells isolated from BALB/c spleen were analysed by flow cytometry after 8 days of culture. Two representative profiles (left column, middle column) of viable CD19^+^ B cells stained for CD38, FAS(CD95), GL7, CD138 and intracellular IgG1 and IgM, or with isotype control monoclonal antibody (right column) are presented.

Supplementary Figure 4 Efficacy of single-cell iGB-culture from FACS-sorted B cells from spleen NTL mice, and from cervical lymph nodes and brain of disease-free- and diseased TCR^1640^ mice. **(A)** Percentage of wells containing iGB clusters and **(B)** mean expansion fold of iGB clusters after 8 to 12 days of single-cell iGB culture.

Supplementary Figure 5 Distance tree of NCBI protein blasted with mus musculus (taxid:10090) as target organism and the short consensual sequence CQQWSSYPLT as probe (Fast Minim Evolution, 0.90 of max sequence difference, distance of Grishin for proteins).
