# Supplementary figures and images for: A Spontaneous Model of Experimental Autoimmune Encephalomyelitis Provides Evidence of MOG-Specific B Cell Recruitment and Clonal Expansion
Source: Front Immunol. 2022 Feb 3;13:755900. doi: 10.3389/fimmu.2022.755900 (PMC8850296; doi:10.3389/fimmu.2022.755900)

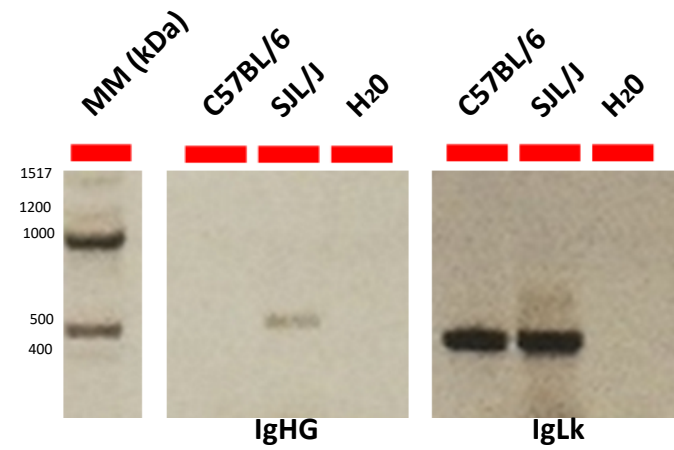

50 microg 8-18C5 (n=4)

PBS (n=4)

Serum NTL (n=7)

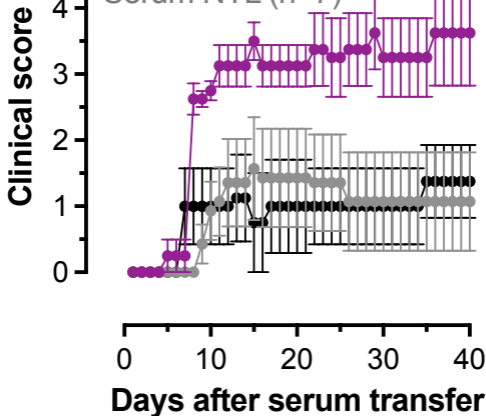

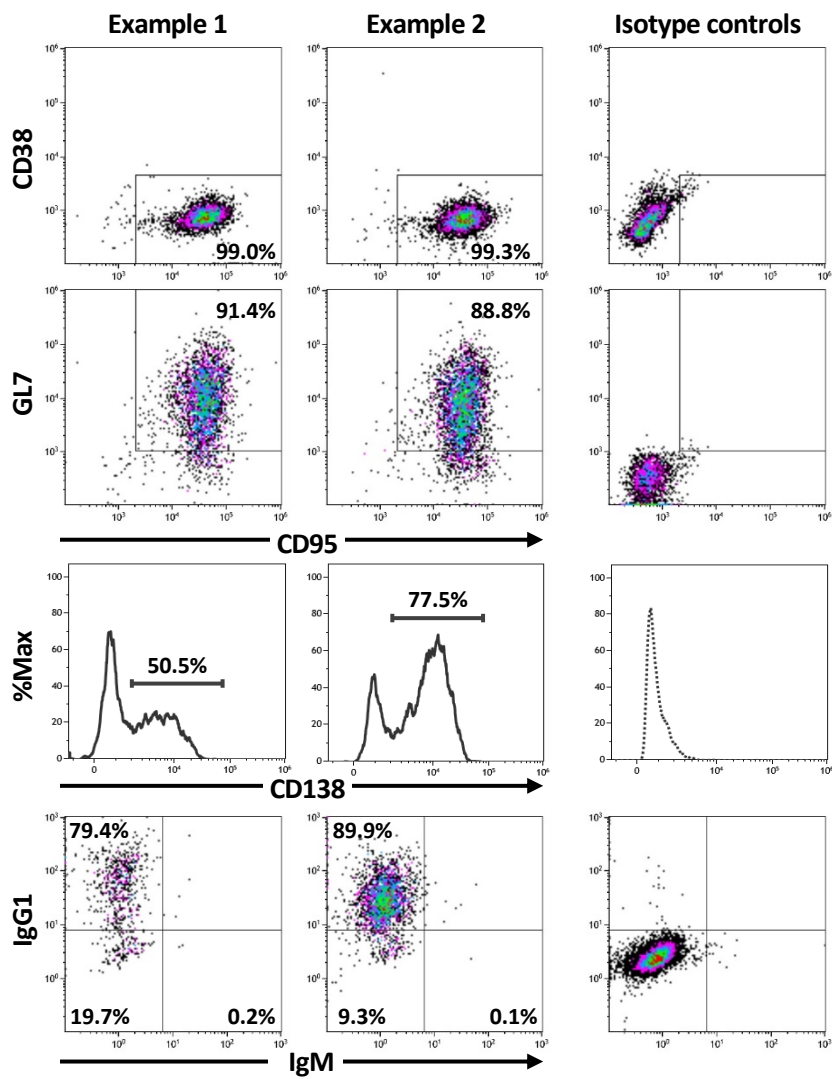

**A**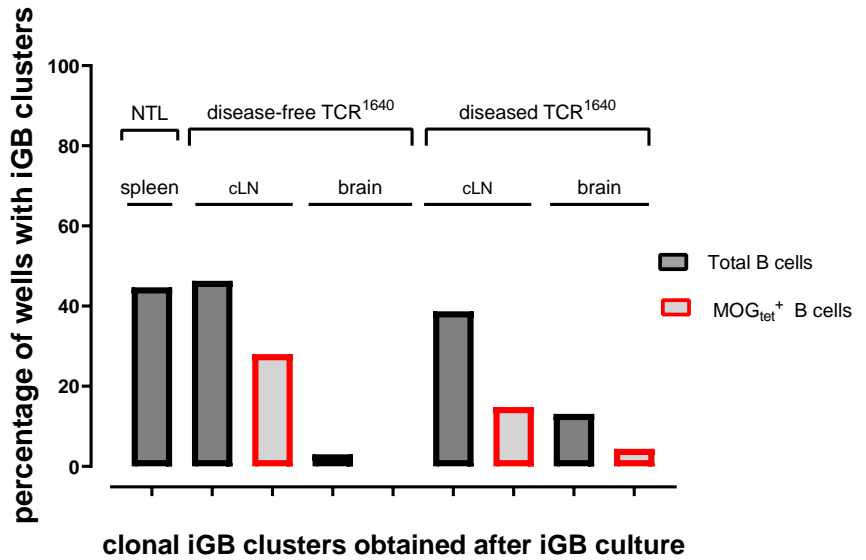**B**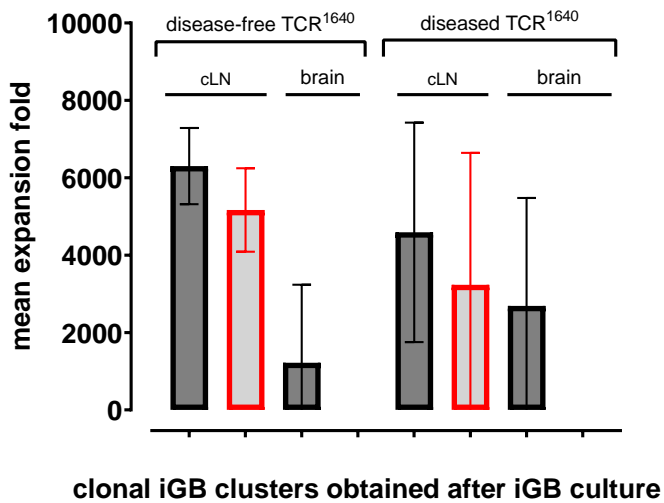

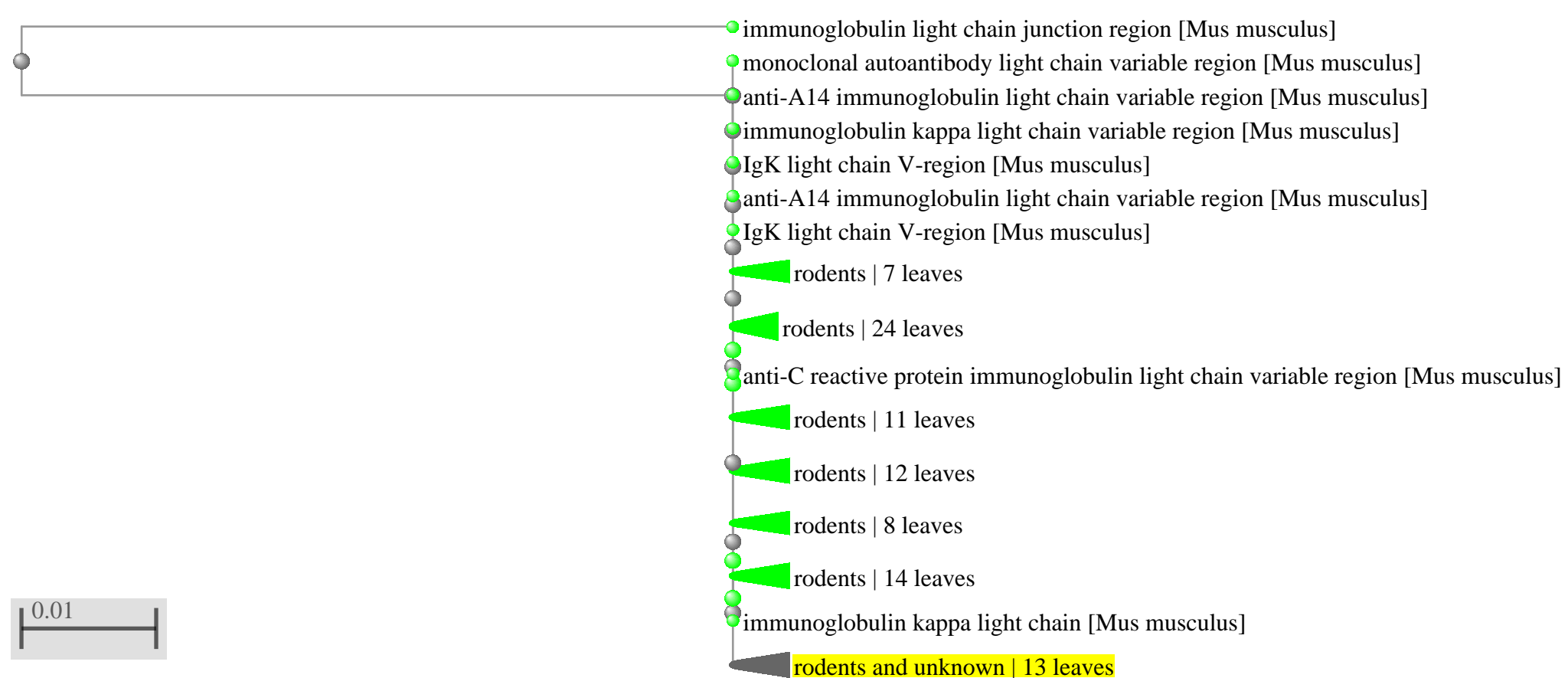

Supplement: Supplementary Figure 1 — Amplification of the antibody variable region. Validation of the primer pairs presented in Table 1 . cDNA was obtained from purified B cells/iGB cells of SJL/J and C57BL/6 mice. Amplified PCR products migrated to expected molecular mass size (close to 500 kDa for IgHG and close to 400 kDa for Igk). Sequencing of the purified amplicon for SJL/J mice confirmed the amplification of the hypervariable regions of murine IgHG and Igk chains (data not shown). [file DataSheet_1.pdf]
