# Supplementary material for: A Spontaneous Model of Experimental Autoimmune Encephalomyelitis Provides Evidence of MOG-Specific B Cell Recruitment and Clonal Expansion
Source: Front Immunol. 2022 Feb 3;13:755900. doi: 10.3389/fimmu.2022.755900 (PMC8850296; doi:10.3389/fimmu.2022.755900)
Supplement: Supplementary file 2 [file Table_1.docx]

| **Supplementary Table 1.** Primers designed for variable parts of heavy G and light kappa chains of Immunoglobulins in B cells from SJL/j mice | | |
| --- | --- | --- |
|  |  | 5'3' |
| IgH forward |  | saggtscagctgcagsagtgtgg |
| IgHG reverse |  | ctcaggggaartavccyttgac |
| IgLK forward |  | gayattgtgmtsacmcarwctmca |
| IgLK reverse |  | gatggtgggaagatacagtt |
|  |  |  |
| Ig: Immunoglobulin; H: heavy; L: light; k: kappa | | |
